# Treatment of Oral Biofilms by a D-Enantiomeric Peptide

**DOI:** 10.1371/journal.pone.0166997

**Published:** 2016-11-23

**Authors:** Tian Zhang, Zhejun Wang, Robert E. W. Hancock, César de la Fuente-Núñez, Markus Haapasalo

**Affiliations:** 1 Division of Endodontics, Department of Oral Biological and Medical Sciences, Faculty of Dentistry, University of British Columbia, Vancouver, British Columbia, Canada; 2 Centre for Microbial Diseases and Immunity Research, Department of Microbiology and Immunology, University of British Columbia, Vancouver, British Columbia, Canada; 3 Synthetic Biology Group, MIT Synthetic Biology Center, Research Laboratory of Electronics, Department of Biological Engineering, Department of Electrical Engineering and Computer Science, Massachusetts Institute of Technology, Broad Institute of MIT and Harvard University, Cambridge, Massachusetts, United States of America; Nanyang Technological University, SINGAPORE

## Abstract

Almost all dental diseases are caused by biofilms that consist of multispecies communities. DJK-5, which is a short D-enantiomeric, protease-resistant peptide with broad-spectrum anti-biofilm activity, was tested for its effect on oral multispecies biofilms. Peptide DJK-5 at 10 μg/mL effectively prevented the growth of these microbes in culture media in a time-dependent manner. In addition to the prevention of growth, peptide DJK-5 completely killed both *Streptococcus mutans* and *Enterococcus faecalis* suspended from biofilms after 30 minutes of incubation in liquid culture media. DJK-5 also led to the effective killing of microbes in plaque biofilm. The proportion of bacterial cells killed by 10 μg/mL of DJK-5 was similar after 1 and 3 days, both exceeding 85%. DJK-5 was able to significantly prevent biofilm formation over 3 days (*P* = 0.000). After 72 hours of exposure, DJK-5 significantly reduced and almost completely prevented plaque biofilm production by more than 90% of biovolume compared to untreated controls (*P* = 0.000). The proportion of dead biofilm bacteria at the 10 μg/mL DJK-5 concentration was similar for 1- and 3-day-old biofilms, whereby >86% of the bacteria were killed. DJK-5 was also able to kill >79% and >85% of bacteria, respectively, after one-time and three brief treatments of 3-day-old biofilms. The combination of DJK-5 and chlorhexidine showed the best bacterial killing among all treatments, with ~83% and >88% of bacterial cells killed after 1 and 3 minutes, respectively. No significant difference was found in the percentage of biofilm killing amongst three donor plaque samples after DJK-5 treatment. In particular, DJK-5 showed strong performance in inhibiting biofilm development and eradicating pre-formed oral biofilms compared to L-enantiomeric peptide 1018. DJK-5 was very effective against oral biofilms when used alone or combined with chlorhexidine, and may be a promising agent for use in oral anti-biofilm strategies in the future.

## Introduction

Despite the best efforts of dental health professionals, oral infections are still widespread. Nearly 85% of North American adults between the ages of 20 and 64 have dental restorations, and 23.7% of them have untreated dental caries [1]. More than 47% of American adults have mild, moderate, or severe periodontitis [2]. Recent molecular methods have revealed that almost all dental diseases are caused by dental biofilms that consist of multispecies microbial communities [3–6]. Oral microbial biofilms are three-dimensionally structured communities embedded in an exopolysaccharide matrix [7–10] attached to solid surfaces such as tooth enamel, the surface of the root or dental implants [11], and they are extremely difficult to treat [10]. The most notable difference between oral bacteria in dental biofilms and the same strain grown planktonically is the increased tolerance/adaptive resistance of mature biofilm bacteria to antimicrobial agents. According to Sedlacek and Walker [12], the concentration of the antibiotic required to inhibit the growth of bacterial strains in biofilms is approximately 250 times greater than when the same strains are grown planktonically.

Cationic host defense peptides and their synthetic derivatives (innate defense regulators) have been proposed to be alternative approach in the treatment of infections [13]. There are more than 2,100 host defense peptides (also termed antimicrobial peptides) in nature and these collectively have very broad activities including partially independent immunomodulatory, direct antimicrobial, and anti-biofilm activities [14–16]. However, one major obstacle to their success as therapeutics in clinical trials is their inherent susceptibility to proteolytic degradation [17–19]. Researchers have attempted to solve some of these limitations by physicochemical modifications to the peptides. A potentially effective way to improve the proteolytic stability of peptides is to incorporate non-natural D-isomers of amino acids, which change the stereochemistry of the peptides making them more resistant to proteases. The peptide with D-isomers can maintain the antimicrobial activity of the native sequence because these peptides interact directly with, and often translocate across, the bacterial membrane rather than requiring a specific receptor [20–22]. Recently, a broad-spectrum L-amino-acid-containing peptide (1018) was shown to act against biofilm microbes by triggering the degradation of guanosine tetraphosphate (ppGpp) [23], which is important in biofilm development of many bacterial species [24]. Peptide 1018 was also shown to inhibit oral plaque biofilm formation and to have antimicrobial activity against oral plaque biofilms [25]. DJK-5, a D-enantiomeric protease-resistant 12-amino-acid peptide, was also shown to have broad-spectrum anti-biofilm activity through a similar mechanism to 1018 (promoting ppGpp degradation) [24, 26]. To date, there have been no studies on DJK-5 to assess whether the peptide might be suitable for use in dental settings. Therefore, the objective of this project was to examine the effectiveness of the new antimicrobial peptide, DJK-5, against oral plaque biofilms from different sources. Furthermore, it was also studied in combination with another cationic agent, chlorhexidine (CHX).

## Materials and Methods

### Peptide Synthesis

Peptides 1018 and DJK-5 were synthesized by CPC Scientific (Sunnyvale, CA, USA) using solid-phase 9-fluorenylmethoxy carbonyl (Fmoc) chemistry and purified to a purity of >95% using reverse-phase high-performance liquid chromatography as previously described [22]. For the experiments the peptide was taken from peptide stocks in deionized water. The sterility of the peptide stocks was checked.

### Plaque Bacteria Isolation and Measurement of Minimal Inhibitory Concentration

The study was approved by the University of British Columbia Clinical Research Ethics Board (certificate H12-02430). Written informed consent was obtained from the participants for collecting the saliva and plaque bacteria in this study [25]. Plaque samples were collected with sterile wooden toothpicks from three volunteers and grown overnight in the specified medium [i.e., brain heart infusion (BHI) or Luria-Bertani (LB) broth] [25]. Bacteria were grown under anaerobic conditions at 37°C using anaerobic bags. The OD values of the plaque samples collected at the beginning of bacterial culture for minimal inhibitory concentration assays were 0.10 for BHI medium and 0.05 for LB medium. Overnight cultures were diluted 1:100 in their growth medium and transferred to 96-well plates containing increasing concentrations (0, 5, 10, 20, 40, and 80 μg/mL) of peptide DJK-5 in a biological safety cabinet (VWR Inc., Edmonton, Alberta, Canada) at room temperature. After 24 hours of peptide treatment under anaerobic condition at 37°C, bacterial growth was measured at an absorbance of 630 nm.

### Biofilm Model

Biofilms were grown on sterile hydroxyapatite (HA) disks (Clarkson Chromatography Products, Williamsport, PA, USA) as previously described [25]. A saliva-coated HA disk (sHA) was prepared by incubating each HA disk in the wells with saliva that was collected from three volunteers. Supra- and subgingival plaque was collected from each of three healthy adult volunteers. Only one donor’s plaque bacteria were tested for inhibition by peptide 1018, while all 3 were used for DJK5. The sHA disks were incubated under anaerobic conditions at 37°C for 3 days [25].

### Biofilm Inhibition Test

Peptide 1018 or DJK-5 at two different concentrations (10 and 5 μg/mL, corresponding to 6.5 and 3.25 μM, respectively) was added to the plaque suspension at the beginning of biofilm development, and grown anaerobically at 37°C for up to 3 days [25]. For the control group, sterile water was added into the culture medium instead of peptide. Three sHA disks were included in each group at each time interval.

### Long-term Anti-biofilm Effect of Peptides on Pre-formed Biofilms

Using the method described in the biofilm inhibition test, 9 biofilm-covered sHA disks (3-day-old biofilm) per group were exposed to the peptide at the two different concentrations (10 and 5 μg/mL) in BHI. Three sHA disks for each peptide concentration were incubated with the peptide for 24 hours under anaerobic conditions at 37°C (24-hour treatment) [25]. Another three sHA disks were similarly incubated for another 24 hours with the same concentration of the peptide solution (48-hour treatment), and the remaining three disks in each group were treated a third time and incubated for a third 24-hour period (72-hour treatment). Control specimens with no peptide (only BHI + sterile water) were included for each time period (24, 48, and 72 hours).

### Short-term Anti-biofilm Effect of Peptides on Pre-formed Biofilms

Short term exposure to the peptides was performed as previously described [25]. Briefly, twelve 3-day-old plaque biofilm sHA disks were rinsed in phosphate buffered saline (PBS) at pH 7.0 for 1 minute. Disks were then immersed in 1 mL of 10 μg/mL (6.5 μM) of peptide 1018 or DJK-5 for 1 or 3 minutes for either one or three treatments. Six disks treated with sterile water were used as the negative control group. Disks treated three times with the peptide were immersed in PBS for 1 minute between each treatment.

### Anti-biofilm Effect of Peptides in Combination with CHX

Three-day-old plaque biofilm disks were prepared and rinsed in PBS for 1 minute. The disks were then divided into six treatment groups: (i) sterile water, (ii) 2% CHX, (iii) 10 μg/mL (6.5 μM) of DJK-5, (iv) 10 μg/mL (6.5 μM) of 1018, (v) 2% CHX+ 10 μg/mL (6.5 μM) of DJK-5, and (vi) 2% CHX+ 10 μg/mL (6.5 μM) of 1018. The sHA disks were immersed in 2-mL solutions of each medicament for 1 or 3 minutes.

### Confocal Laser Scanning Microscopic Examination of Biofilm Samples Untreated or Treated with Peptides and/or CHX

The biofilms exposed to the different treatments were examined by viability staining and confocal laser scanning microscopy as previously described [25]. Biofilm disks were rinsed in 0.85% physiological saline for 1 minute before staining. LIVE/DEAD Bac-Light Bacterial Viability kit L-7012 for microscopy and quantitative assays (Molecular Probes, Eugene, OR, USA), containing two component dyes (SYTO 9 and propidium iodide in a 1:1 mixture) in solution, was used for staining the biofilm following the manufacturer’s instructions. The excitation/emission wavelengths were 480/500 nm for SYTO 9 and 490/635 nm for propidium iodide for collecting the fluorescence. Fluorescence from each stained cell was viewed using a confocal laser scanning microscope (FV10i-LIV, Olympus, Canada) equipped with 4 laser diodes (405nm, 473nm, 559nm, 635nm) at a 1024 × 1024 pixel scan area using a 10 × lens. Four random areas of the biofilm on each disk were scanned. A stack of 80–100 slices in 0.5 μm step sizes was captured from the top to the bottom of the biofilm. Confocal images were then quantitated and analyzed (live/dead ratios) using Imaris 7.2 software (Bitplane Inc., St. Paul, MN, USA). The thresholds were set manually by adjusting the gain sensitivity of the red and green fluorescence respectively to a level just below overexposure of areas in the phase contrast mode. The thresholds were set by adjusting the gain sensitivity of the red and green fluorescence respectively to a level where overexposure spots just disappeared in the phase contrast mode. The absolute threshold value was the same for all the four scans for each sample but might be different for different samples in order to avoid over- and underexposure of fluorescence. The original confocal data was uploaded to Imaris 7.2 software and the intensity of green and red fluorescence in full thickness of biofilm layers were captured automatically. The software reconstructed the 2-dimentional intensity of fluorescence in all the layers to a 3-dimentional volume stack. The volumes for each fluorescence were then calculated and the proportion of dead bacteria was calculated as follows: Dead bacteria% = (red fluorescence volume) / (green fluorescence + red fluorescence volume) × 100% [8,25].

### Effect of Peptide DJK-5 on the Growth of Bacteria Suspended from Biofilms into BHI or LB Broth Culture

*Enterococcus faecalis* VP3-181, *Streptococcus mutans* NCTC 10449, and three plaque samples collected from three healthy volunteers were used for the broth culture experiment. The sHA disks were placed into the 24-well plates, each containing 1.8 mL of BHI or LB. Each well was inoculated with 0.2 mL of dispersed bacterial suspension (*E*. *faecalis*, *S*. *mutans*, and plaque) [25]. Biofilm growth, preparation of bacterial suspensions and killing experiments were as previously described [25]. Briefly, after three days of incubation anaerobically (plaque and *S*. *mutans*) or aerobically (*E*. *faecalis*) the biofilms were scraped off into BHI or LB medium and dispersed by pipetting and vortexing. The suspensions were adjusted to an OD_405_ of 0.25 for the three microbe groups and a 100-μL sample of the bacteria in suspension was added to 400 μL of 8 μg/mL (5.2 μM) peptide DJK-5 in BHI or LB for 0, 30, 60, or 120 minutes. BHI or LB alone was used as the negative control for each time interval. After each exposure, 100 μL of bacterial solution was added to 900 μL of corresponding culture medium, serially diluted and spotted onto blood agar plates for counting the colony forming units (CFU). The plates were incubated anaerobically (*S*. *mutans* and plaque) or aerobically (*E*. *faecalis*), at 37°C for 72 hours and the Log_10_CFU count was calculated. Three independent experiments were performed with three replicates each [25].

### Statistical Analysis

Statistical analysis was performed with SPSS 16.0 software for Windows (SPSS, Chicago, IL, USA). Homogeneity of variance was determined using Levene’s test. Univariate ANOVA was applied and post hoc multiple comparisons were used to isolate and compare the results (mean ± standard deviation) at the *P* < 0.05 significance level.

## Results

**Fig 1:**
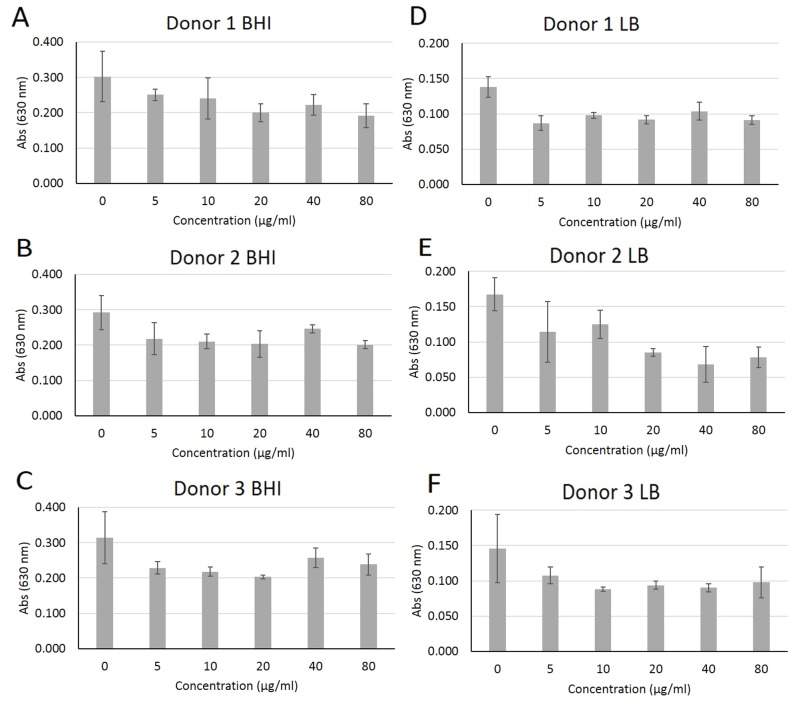
Modest effect after 24 hours of increasing concentrations of peptide DJK-5 on the planktonic growth of plaque bacteria grown in BHI or LB medium. Bacteria from plaque samples were grown in BHI and LB medium using 96-well polypropylene plates in the presence of increasing concentrations of peptide DJK-5 and planktonic growth (measured absorbance at 630 nm) was assessed after 24 hours. Error bars represent the standard deviations calculated from three independent experiments.

Peptide DJK-5 treatment did not substantially affect the planktonic growth of plaque bacterial communities (Fig 1). When grown in BHI, DJK-5 only modestly reduced growth for the three individual samples tested. Similar results were obtained when plaque bacteria were grown planktonically in LB medium in the presence of the peptide, although the peptide was able to significantly inhibit the growth of plaque from donor 2 (*P* = 0.001) at 40 μg/mL (26 μM) by about 50%.

**Fig 2:**
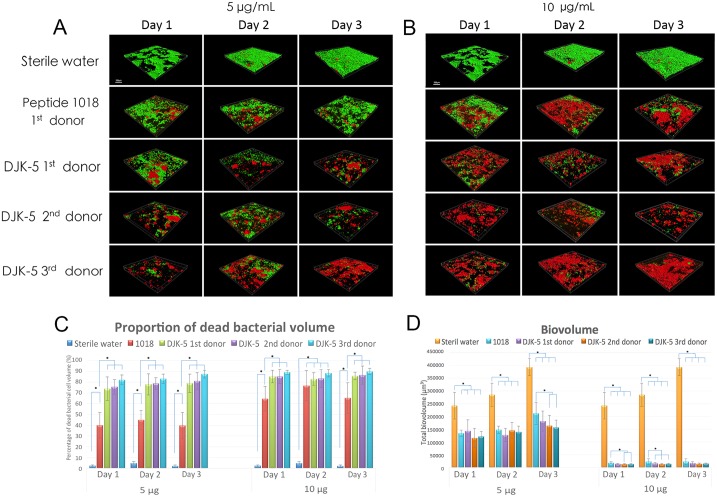
Peptide DJK-5 had a greater effect than 1018 on oral multispecies biofilm development. (A, B) Confocal microscopy images of plaque biofilm development on hydroxyapatite-coated disks over 3 days in the presence of (A) 5 μg/mL and (B) 10 μg/mL peptide or water as a control. The scale bar represents 200 μm. (C) The proportion of dead biofilm bacterial cell volume during 3-day biofilm development in the presence of peptides 1018 and DJK-5. (D) Total biovolume of plaque biofilm formed in 3 days in the presence of peptides. Error bars represent the standard deviations calculated from three independent experiments.

For studying the ability of DJK5 to inhibit biofilm bacteria, 1018 was used as a positive control [25]. As described below, in all instances DJK5 was superior to 1018 in its killing efficacy, which might have been due to its resistance to the proteolytic activity of plaque biofilm bacteria. In the biofilm growth inhibition test, significantly more microorganisms were killed at the higher concentration (10 μg/mL, 6.5 μM) of DJK-5 than at the lower concentration (5 μg/mL, 3.25 μM) (Fig 2; *P* = 0.004) as shown previously for the positive control peptide 1018 [25] (Fig 2). Notably, DJK-5 showed much stronger effects than 1018 in killing significantly more biofilm microbes at both concentrations (*P* < 0.01). There was no significant difference in the proportion of killed bacteria between 1-, 2-, and 3-day-old biofilms by each of the peptides and the proportion of bacteria killed by DJK-5 at 10 μg/mL (6.5 μM) at days 1 and 3 exceeded 85% (Fig 2C). It is important to note that DJK-5 showed a similar killing pattern for the plaque biofilms derived from the three different donors with virtually no killed bacteria in the negative controls (water-treated; *P* = 0.004). Exposure to high concentrations of peptide DJK-5 during biofilm growth reduced the plaque biofilm biovolume by more than 10-fold after 72 hours of treatment (*P* = 0.000), resulting in only 8%, 8%, and 6% after 24-, 48-, and 72-hour time intervals, respectively, compared to control biofilm (Fig 2D).

**Fig 3:**
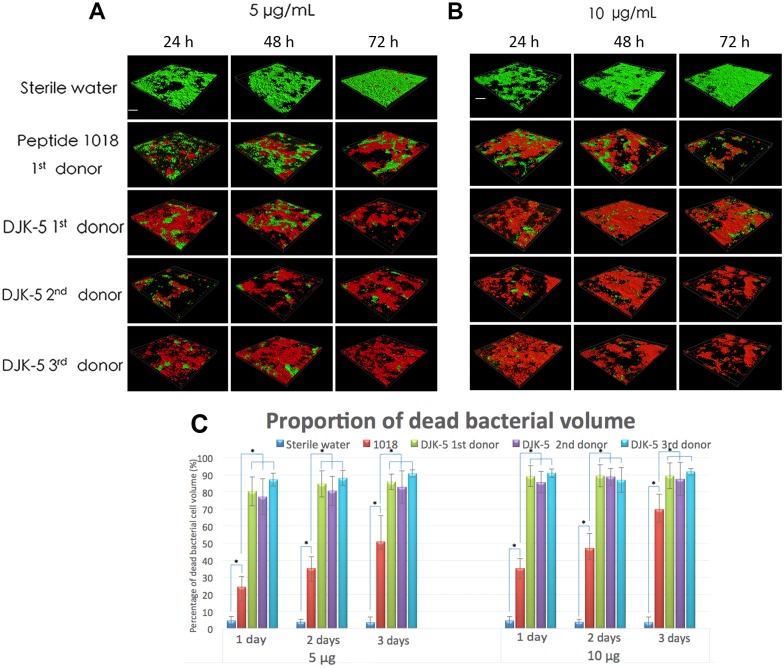
Killing of microbes in 3-day-old biofilms exposed to the peptides for 24, 48, and 72 hours. (A, B) Confocal microscopy images of 3-day-old plaque biofilms treated with (A) 5 μg/mL or (B) 10 μg/mL of peptides 1018 and DJK-5. Left column in (A) and (B): samples treated once were challenged with peptide for 24 hours after biofilm formation for 3 days. Middle column in (A) and (B): biofilms treated twice were exposed to peptide after day 3 for one more day (48 hours). Right column in (A) and (B): biofilms treated three times were exposed to the peptide for two more days (72 hours). The scale bar in (A) and (B) represents 200 μm. (C) The proportion of dead biofilm bacterial cell volume after exposure to the peptides for 24, 48, and 72 hours. Error bars represent the standard deviations calculated from three independent experiments.

Exposure of pre-formed biofilms to DJK5 after 3 days of biofilm growth showed similar results as obtained when the biofilms were exposed to the peptides from the beginning of growth (Fig 3). Again, DJK-5 was considerably more effective in killing biofilm microbes than the positive control peptide 1018 at both concentrations (Fig 3; *P* = 0.001). The number of killed biofilm bacteria was significantly correlated with the concentration of peptide used (*P* < 0.05). The proportion of dead bacterial cells at the higher DJK-5 concentration (10 μg/mL, 6.5 μM) was similar between 1 day (85–91%) and 3 days (87–91%) in all three donors’ biofilms and in all cases (Fig 3C).

**Fig 4:**
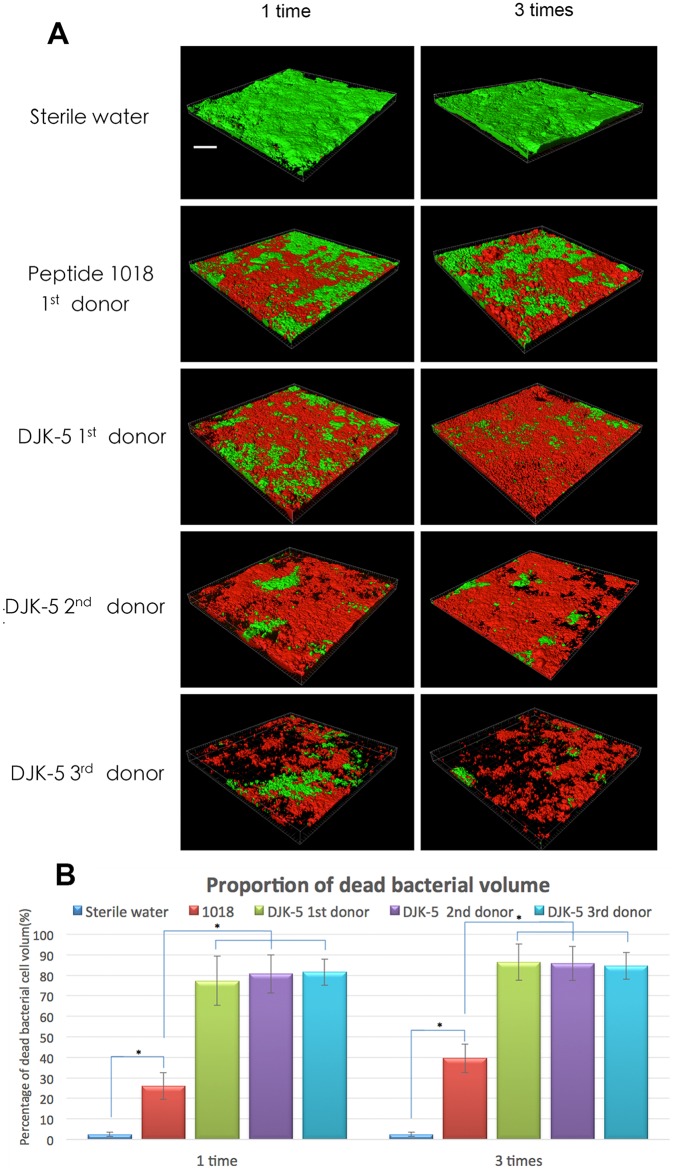
Effect of brief exposure to anti-biofilm peptides on pre-formed plaque biofilms: superior efficacy of DJK-5. (A) CLSM images of 3-day-old plaque biofilms after one or three 3-minute exposures to the peptides (10 μg/mL). The scale bar represents 200 μm. (B) The proportion of dead bacterial cells after different peptide treatments for 3 minutes. Error bars represent the standard deviations calculated from three independent experiments.

In short exposures of pre-formed biofilms, the proportion of dead bacterial cells was significantly correlated with the agent of exposure and the number of repeated medicament applications (Fig 4). Peptide DJK-5 at 10 μg/mL (6.5 μM) applied for 3 minutes on 3-day-old biofilms once or 3 times killed a much higher percentage of plaque biofilm bacteria (77–86%) than did the positive control peptide 1018 (26–40%; *P* = 0.000). Again, no significant difference was found in the sensitivity of biofilms from the three donors to peptide DJK-5.

**Fig 5:**
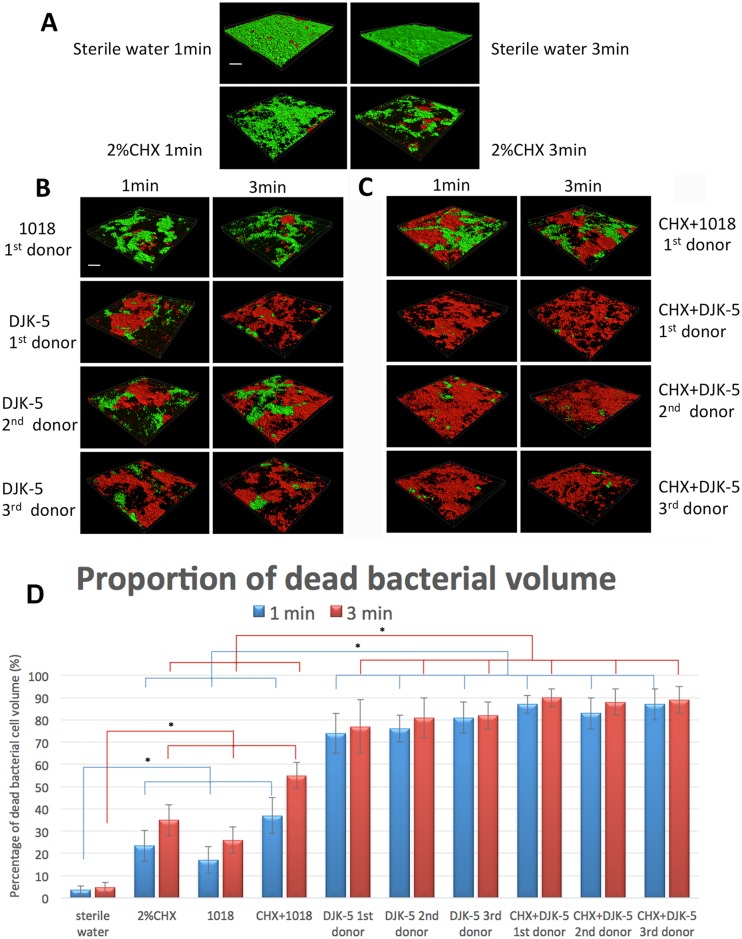
Superior killing of plaque biofilm microbes by peptide DJK-5 (cf. 1018) with chlorhexidine when used in combination for 1 or 3 minutes. (A-C) 3D CLSM images; the scale bar represents 200 μm. (D) The proportion of dead microbial cells after treatment with peptide, CHX, or the combination of both agents. Data calculated from A-C. Error bars represent the standard deviations calculated from three independent experiments.

The combination of DJK-5 and CHX showed the best results in bacterial killing among all groups. The combination killed 83–87% and 88–90% of the bacteria after just 1 and 3 minutes, respectively (Fig 5; *P* = 0.000). DJK-5 alone killed a much higher percentage of bacteria than the combination of 1018 and CHX, or either CHX or peptide 1018 alone (*P* < 0.01).

**Fig 6:**
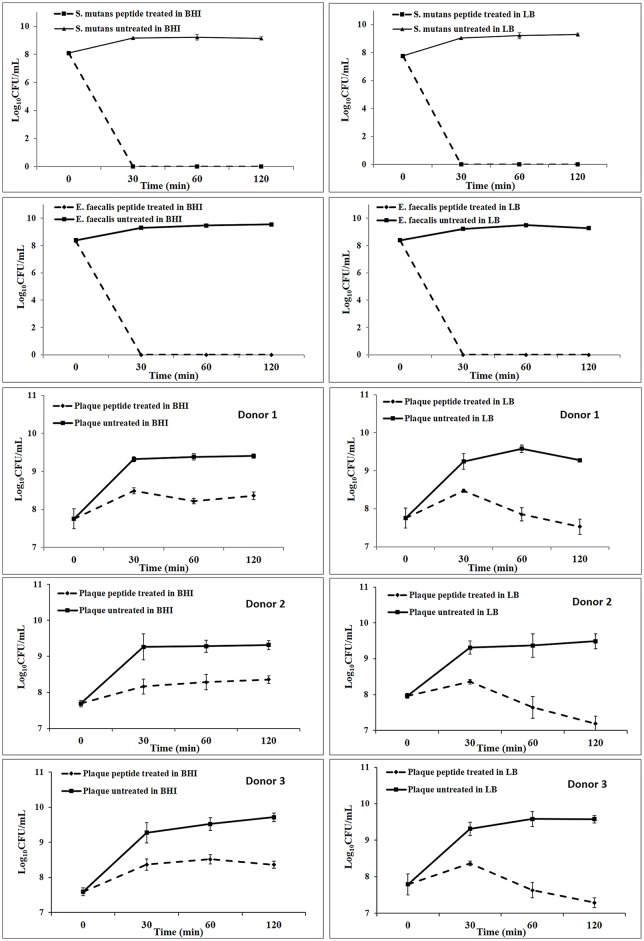
Growth curves of dental plaque, *S*. *mutans*, and *E*. *faecalis* suspended from sHA biofilms into BHI or LB broth containing 400 μL of 8 μg/mL DJK-5 peptide. The concentrations of viable bacteria were measured by performing CFU counts over time after resuspension of the biofilms. Error bars represent the standard deviations calculated from three independent experiments.

The effect of peptide DJK-5 on bacteria suspended from 3-day-old biofilms into liquid culture in BHI or LB was examined over a period of 120 minutes (Fig 6). Peptide DJK-5 at 10 μg/mL (6.5 μM) effectively prevented the growth of microbes resuspended from biofilms into both culture media in a time-dependent manner. In addition to the prevention of growth, peptide DJK-5 completely killed *S*. *mutans* and *E*. *faecalis* after 30 minutes of incubation in both culture media. DJK-5 also led to very effective killing of microbes from the plaque biofilm with between 99 and 100% of the microbes being dead after 30 minutes. Plaque bacteria in LB medium were modestly more susceptible to the action of the peptide killing than in BHI medium, which might have been due to the increased nutrient content of BHI [27] that suppressed the formation of ppGpp, the target of DJK5. However, there is no statistically significant difference in CFU numbers at different time intervals between LB and BHI (*P* = 0.085) (Fig 6).

## Discussion

Bacterial resistance strategies to antimicrobial peptides that include the enzymatic degradation of L-enantiomeric peptides have been described previously [28,29], and host proteases can also degrade such peptides during therapy. D-enantiomeric and retroinverso 12-mer peptides based on the physicochemical properties of active anti-biofilm peptides were recently designed [24,26]. As we have previously described, only 9 of the 20 natural amino acids (V, R, L, I, A, W, F, K, Q) are included in the new designs 4 charged residues (most commonly R), 7 or 8 hydrophobic residues, and no more than 1 glutamine (Q). D-amino acids are not naturally present in human peptides and therefore make them resistant to degradation by host proteases [30]. This is especially important in the oral cavity where most organisms produce secreted proteases [31]. Both the D-amino acid peptide DJK-5 and the L-amino acid peptide 1018 promote ppGpp degradation; in addition, the D-amino acid peptides have enhanced biological activities *in vitro* [23,24]. DJK-5 was one of several recently designed peptides [30] which showed enhanced, broad-spectrum anti-biofilm activity when tested on several species of Gram-negative pathogens [24,26]. According to de la Fuente-Núñez [24], D-enantiomeric peptides are designed not to be recognized by bacterial or host proteases that abound during infections. They showed additive or synergistic effects with conventional antibiotics, rendering biofilms more susceptible to these agents [24]. Human oral biofilms exist as multispecies communities, which can be isolated and reconstructed *in vitro* in saliva-based model biofilms [32]. Plaque biofilms are known to contain both Gram-negative and Gram-positive species [33]. In the present study, DJK-5 showed excellent performance in inhibiting oral biofilm growth and in eradicating pre-formed oral biofilms, being considerably more potent than peptide 1018. In the current study, DJK-5 displayed strong anti-biofilm activity and was able to kill most oral bacteria in a short time of only 1 or 3 minutes.

Dental caries is a chronic disease of microbiological origin, and *S*. *mutans* has been implicated as one of the major etiological pathogens responsible for the majority of caries [34,35]. *E*. *faecalis* has gained attention in the endodontic literature because it can frequently be isolated from root canals in cases of failed root canal treatment [36,37]. *S*. *mutans* and *E*. *faecalis* are both facultative anaerobes. In order to have equally long time of growth, *S*. *mutans* was cultured anaerobically and *E*. *faecalis* was grown in the air. Our CFU determinations on biofilm bacteria showed that DJK-5 had strong antimicrobial ability in single-species and oral multispecies biofilms. Data from 16S rRNA gene cloning and sequencing studies indicate that the oral cavity has roughly 700 microbial phylotypes, of which slightly less than half have been cultivated [38].

Viability staining, which might better reflect the true viability of oral biofilm bacteria than culturing during some growth phases [39], further confirmed the effectiveness of DJK-5 both with regard to reduction in biofilm biovolume and in direct killing of the bacteria. Viability staining is based on the principle that the red stain (propidium iodide) enters only those cells where the cell membrane is damaged, whereas the green stain (SYTO 9) can enter all cells. It is therefore possible that in some cases red fluorescence may give a false-positive result when interpreted as a killed cell, although the cell is still alive although damaged. Despite its shortcomings, viability staining has become the method of choice in measuring biofilm killing [8,25,39]. The methodology allows for the measurement of the relative proportion of damaged/killed bacteria in each biofilm specimen, which is not possible by using traditional culture methods [39].

With the complexity of multispecies microbial communities, it is difficult to determine the functions of individual species contributing to the observed physiological changes. Biofilms are structured microbial communities embedded in extracellular polymeric substances, and engaged in a distinct lifestyle accompanied by specific gene expression profiles. The extracellular polymeric substance matrix provides several functional purposes for the biofilm, such as protecting bacteria from environmental stresses and providing mechanical stability [40,41]. The multispecies biofilms on sHA grown from plaque from three different donors showed no differences in their susceptibility to either DJK-5 or the combination of DJK-5 and 2% CHX. This result might indicate that the source and possible differences in the species composition of these multispecies biofilms had no major impact on their susceptibility to these agents, which is in accordance with a previous study [42]. Previous studies [42,43] showed that mature multispecies biofilms grown on sHA disks from different donors for 3 weeks were substantially more resistant to disinfection than younger 1- and 2-week-old biofilms. Biofilms from six different sources showed a similar, time-dependent susceptibility pattern [43]. However, in our studies, the specific bacterial species present in the different inocula from dental plaque samples and the residual bacteria after peptide treatment were not identified. Our future studies will focus on identifying these species and exploring the metabolic diversity and peptide susceptibility of oral multispecies biofilms.

Chlorhexidine is an active ingredient in many mouthwash products used to reduce dental plaque and planktonic oral bacteria. The antimicrobial action of CHX is non-specific, and while it effectively prevents plaque growth on a clean tooth surface, it is less effective against established oral biofilms. In addition, CHX has a bitter taste and tendency for staining the teeth [44,45]. Previous study [25] and current results have shown that peptide 1018 is compatible with and can act in combination with CHX to attack established plaque biofilms. In the present study, the combined use of DJK-5 and CHX showed a strong additive effect in bacterial killing. Although DJK-5 alone worked quite well, the combination of DJK-5 and CHX had the highest anti-biofilm effect in short-term exposures against oral multispecies biofilms, when compared to DJK-5 alone or peptide 1018 with or without CHX. Based on these experiments, we anticipate that DJK-5 could have potential for use in daily oral care and/or rapid disinfection during oral surgery.

In conclusion, peptide DJK-5 showed strong performance in inhibiting biofilm growth, eradicated pre-formed oral biofilms, and showed efficient anti-biofilm activity in short-term exposures of 1 and 3 minutes. DJK-5 was more effective against oral biofilms than peptide 1018 when used alone or together with CHX, and may be a promising agent for use in oral anti-biofilm strategies in the future.

## Supporting Information

S1 Appendix Dataset for all experiments. (XLSX)
